# Biochemical and Demographical Differences in Atypical vs. Typical Femoral Fractures: A 10-Year Experience Across Two Centers

**DOI:** 10.1155/ije/9376426

**Published:** 2025-09-27

**Authors:** Aongus O'Brolchain, Zander Englebrecht, Richard Steer, Alfred Phillips, Chen-I. Lin

**Affiliations:** ^1^Griffith University, Gold Coast, Queensland, Australia; ^2^Gold Coast Hospital and Health Service, Gold Coast, Queensland, Australia

**Keywords:** ALP, atypical fracture, biochemistry

## Abstract

**Background:** Atypical femoral fractures (AFFs) are rare but serious complications of antiresorptive therapy (ART), frequently misdiagnosed and managed inappropriately in acute care settings. Early recognition remains critical to avoid further harm.

**Objective:** To compare the clinical and biochemical characteristics of patients with AFFs and typical femoral fractures (TFFs) to identify features that may assist in diagnosis at the time of hospitalization.

**Methods:** A retrospective study was conducted across two tertiary hospitals in Queensland, Australia, from 2012 to 2022. Patients presenting with femoral shaft fractures were identified using ICD-10 codes. Fractures were classified radiologically using ASBMR criteria. Clinical characteristics, biochemical results, and discharge medications were extracted from electronic records. Between-group comparisons were performed using appropriate statistical tests.

**Results:** Of 869 identified fractures, 43 AFFs and 101 TFFs were confirmed. Patients with AFFs were more likely to have a prior diagnosis of osteoporosis (97.7% vs. 35.6%, *p* < 0.01), a history of fragility fracture (53.5% vs. 26.7%, *p* < 0.01), and prodromal symptoms (32.6% vs. 3%, *p* < 0.01). Biochemically, AFF patients had significantly lower alkaline phosphatase (median 56 vs. 83 IU/L, *p* < 0.01) and higher 25-hydroxyvitamin D levels (median 86.8 vs. 69.5 nmol/L, *p*=0.01). Nearly one-quarter had ALP < 40 IU/L. Despite this, 51.3% of AFF patients were discharged on continued ART.

**Conclusions:** Patients with AFFs demonstrate distinct clinical and biochemical profiles at the time of hospital presentation, most notably suppressed ALP. These features may serve as diagnostic clues to prevent ongoing exposure to ART. Greater clinical vigilance is needed to ensure appropriate management and to consider alternative diagnoses such as hypophosphatasia in selected patients.

**Summary**

• The predominant osteoporosis treatments are strongly associated with “atypical” femoral fractures.
• The radiological diagnostic criteria are qualitative.
• Many fractures are preventable and are underdiagnosed.
• In search of objective biochemical diagnostic clues, this study sought to compare blood results between patients with typical and atypical fractures at time of hospitalization.

## 1. Introduction

Atypical femoral fractures (AFFs) are a rare complication of antiresorptive therapy (ART) for osteoporosis. Femoral shaft “fragility” fractures are uncommon, and when encountered, typically originate at the weight-bearing medial cortex of the bone and are oblique or spiral in nature [1]. In contrast, AFFs resemble “fatigue” or “stress” fractures observed in various materials in engineering and are associated with wear and age. Stress fractures are observed in the lower limbs of weight-bearing athletes, where tensional forces acting on the longitudinal axis of the bone cause significant mechanical stress. The resultant “material fatigue” fracture lines run perpendicular to these forces, causing a transversely oriented fracture [2]. Distinguishing AFFs from typical femoral fractures (TFFs) is critically important in the management of these patients. Importantly, cessation of ART following AFF is essential, and ongoing treatment with either bisphosphonates or denosumab is contraindicated [3]. Patients with AFF are at risk of contralateral fracture if ART is continued.

The pathophysiology of the “atypical” fractures in elderly, osteoporotic patients and the causative role of ART is controversial, and the exact mechanisms underlying their genesis is not fully elucidated [4]. It has been postulated that “micro” stress fractures occur on the lateral cortex of the femur and that the spontaneous bone healing and repair that would ordinarily occur is inhibited by the suppression of bone turnover evinced by the use of ART [5]. Several bone turnover markers exist, including serum C-terminal telopeptide of type I collagen and serum procollagen type I N-propeptide, but are specialized, and are not routinely measured when patients present with osteoporotic fractures [6]. Alkaline phosphatase (ALP), ubiquitously conserved in almost all living organisms, catalyzes the dephosphorylation of pyrophosphate (which is structurally analogous to bisphosphonates [7]) and pyridoxal-5′-phosphate in vivo [8] and is a marker of bone turnover. Low serum levels of ALP are associated with risk of AFF, and lower levels are observed in AFF patients treated with ART. Furthermore, there is evidence that a serum ALP of < 40 IU/L may be a risk factor for bilateral AFFs and that these patients warrant close surveillance [9].

Some monogenic skeletal dysplasias can cause fractures that are clinically indistinguishable from AFFs, most notably hypophosphatasia (HP) [10]. HP is a rare metabolic disorder caused by deficiency of ALP activity and results in defective bone mineralization [11]. HP can cause unusual femoral fractures that meet the American Society of Bone and Mineral Research (ASBMR) criteria for AFF [12]. Patients with HP are frequently misdiagnosed with osteoporosis and receive ART, which is contraindicated [13]. In patients presenting with AFF, low serum ALP on serial sampling should prompt clinicians to consider the possibility of HP both with and without a history of ART [14].

Establishing the diagnosis of AFF is of paramount importance in patients with osteoporosis, as ongoing administration of ART is contraindicated. AFFs are rare, but the use of ART is becoming more prevalent. In Australia, the number of people prescribed an osteoporosis medication between 2014 and 2018 increased by 50% [15]. There are several established risk factors for the development of both osteoporosis and AFFs, including rheumatological and chronic kidney disease (CKD), use of medications such as proton pump inhibitors (PPIs) [16], and inhaled and oral corticosteroids [4]. Despite this, there are few known biochemical signals available to distinguish AFFs from TFFs and the diagnosis remains radiological. Therefore, it is essential for all physicians prescribing ART to be cognizant of both clinical (prodromal thigh pain) and radiological (periosteal thickening or incomplete fracture) features. This study sought to investigate for potential diagnostic biochemical and diagnostic clues by comparing admission bloods results and clinical characteristics of patients presenting with both typical and AFFs.

## 2. Methods

**Figure 1:**
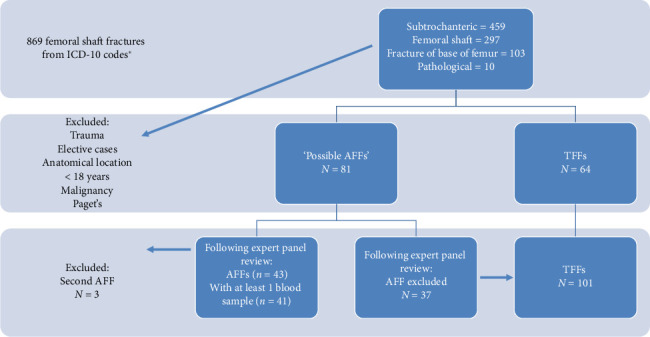
The exclusion process during screening and division of groups by the fracture type. ^∗^ICD-10 codes: M80.45–drug induced osteoporosis with pathological fracture, pelvic region and thigh, fracture of base of neck of femur S72.05, fracture of shaft of femur S72.3, and subtrochanteric fracture S72.2.

The study was conducted at the Gold Coast University and Robina Hospitals on the Gold Coast, Queensland, Australia (ethics approval (LNR) HREC/2022/QGC/84798). Femoral fractures presenting between January 2012 and March 2022 were identified from the electronic medical record using the International Classification of Disease (ICD) codes (M80.45–drug induced osteoporosis with pathological fracture, pelvic region and thigh, fracture of base of neck of femur S72.05, fracture of shaft of femur S72.3, and subtrochanteric fracture S72.2) (see Figure 1).

Radiographs were reviewed by a single reviewer, and all periprosthetic fractures and those proximal to the lesser trochanter and distal to the supracondylar flare (including neck of femur and intertrochanteric fractures) were excluded. Following chart review, all patients with high-energy mechanism of injury along with patients with diseases of bone mineralization (e.g., Paget's disease) or active solid organ or hematological malignancy were excluded. In patients presenting with sequential fractures, only the index presentation was included. Using ASBMR criteria, two consultant orthopedic surgeons and a consultant radiologist performed a blinded review of pelvic and femoral radiographs in random sequence with demographic data removed. Groups were then divided into AFFs and TFFs.

Admission blood results were reviewed and relevant recorded data included lactate, blood group, coagulation profile, alanine aminotransferase (ALT), aspartate aminotransferase (AST), ALP, gamma-glutamyl transferase (GGT), ferritin, creatinine, urea, lactate dehydrogenase (LDH), albumin, thyroid stimulating hormone (TSH), free thyroxine (fT4), and electrolytes (sodium, magnesium, potassium, corrected and ionized calcium, and phosphate). Hemolyzed samples and blood tests collected more than 24 h following presentation were excluded. Serum vitamin B12, folate, and 25-hydroxyvitamin D (25OHD) levels were also recorded, where available, provided this was done within 72 h of admission. Where less than 60% of the patients had blood tests available for a particular parameter, these were excluded to minimize potential bias and unreliable comparisons due to differing patient background.

Demographic information was recorded including age, sex, weight, body mass index (BMI), and length of stay. Clinical characteristics were documented including: the presence of a prodrome prior to the fracture and previous fracture history. Lists of comorbidities (osteoporosis, chronic obstructive pulmonary disease [COPD], rheumatological disease, dementia, CKD, and medication lists (bisphosphonates, denosumab, PPIs, thyroxine, and antidepressants)) were recorded, including duration of ART. Additionally, the prescription of ART at discharge was recorded.

Statistical analysis was performed using Stata17 (College Station, Tx, USA). Means with standard deviations and medians with interquartile ranges were used. For continuous variables with normal distribution, the *t*-test was used (mean with standard deviation (SD)). For non-normally distributed continuous variables, the Mann–Whitney *U* test was used to compare groups by AFF status (median with interquartile range (IQR)). Normality of distribution by assessed using the Shapiro–Wilk test. For categorical variables, Fisher's exact test was used.

## 3. Results

Eight-hundred and sixty-nine femoral shaft fractures were identified using ICD-10 codes. Using ASBMR criteria, 81 possible candidates for AFF were identified. Following expert panel review, a cohort of 46 AFFs was identified. Following chart review and application of exclusion criteria (3 AFFs were sequential and 2 AFFs had no blood tests for inclusion) (Figure 1), 41 AFFs and 101 TFFs had at least one blood sample available for inclusion in the analysis of blood results. The remaining 2 AFFs without blood results were included in the comparison of clinical characteristics. Ferritin, ionized calcium, lactate, folate, activated partial thromboplastin time (APTT), and fibrinogen were excluded from comparison due to results available for fewer than 60% of the patients in each cohort.

**Figure 2:**
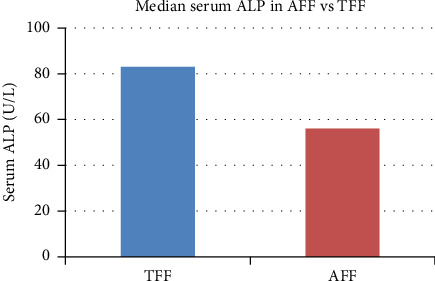
Differences in median serum alkaline phosphatase (ALP) in TFF vs. AFF.

The mean age in years was 73.7 for the AFF group and 76 in the TFF group (*p*=0.04). Serum ALP levels in patients with AFF were significantly lower (Figure 2) and almost a quarter of patients (22.5%) in the AFF group had a serum ALP < 40 IU/L compared with 3.9% (*p* < 0.01) in the TFF group. Significant differences were observed in median serum ALP (56 IU/L vs. 83 IU/L, *p* < 0.01), 25OHD (86.8 nmol/L vs. 69.5 nmol/L, *p*=0.01) (Figure 1), albumin (38 g/L vs. 36 g/L, *p*=0.02), and vitamin B12 (360 pmol/L vs. 249 pmol/L, *p*=0.03). There was no significant difference observed in calcium, phosphate, magnesium, or renal function or other biochemical and hematological parameters (Table 1). There was no significant difference in proportions of blood groups between the two groups (Table 2).

Significant differences between groups were observed in mean weight (64.5 kg [SD 14.9] vs. 72.8 kg [18.4], *p*=0.02), pre-existing diagnosis of osteoporosis (97.7% vs. 35.6%, *p* < 0.01), previous fracture (53.5% vs. 26.7%, *p* < 0.01), and dementia (7% vs. 24.8%, *p* < 0.01). Clinical and fracture characteristics, along with medication history and demographic information of AFF patients are shown in Table 3. In the AFF group, 39.5% (17/43) of the patients were taking 25OHD at admission vs. 35.6% (36/101) in the TFF group, but this was not statistically significant (*p*=0.45).

More than half (22/43, 51.2%) of the patients with AFF were discharged on ART.

Bone biopsy was performed intraoperatively in 7 patients (5 TFFs and 2 AFFs) to exclude pathological fracture. None of the biopsies revealed malignancy or granulomatous disease.

## 4. Discussion

There are minimal data on serum biochemistry in general and ALP concentrations in particular with respect to AFF patients in the hospital setting [9]. This study sought to compare biochemical and hematological data of patients hospitalized with AFF vs. TFFs. Significantly lower levels of ALP were observed in patients presenting with AFF. ALP levels can be transiently depressed for several reasons, including acute illness and recent ART, the latter of which reduces ALP by up to 40% [17]. However, it may also reflect the longer duration of antiresorptive exposure, and a low ALP is an indicator of antiresorptive efficiency in the treatment of osteoporosis [18]. It should be noted from the outset that ALP is nonspecific, and isoforms are synthesized in bone, liver, intestine, and placenta.

Low levels of ALP in the serum may reflect occult metabolic bone disease (such as HP). In a Danish study that examined patients with low ALP presenting to an ambulatory endocrinology clinic over 12 years, more than half of the patients who underwent genetic testing were confirmed to have HP [19]. In a recent Australian study, all patients presenting to osteoporosis clinic with persistently low ALP levels who underwent genetic testing were diagnosed with HP [14]. A systematic review concluded that the rate of AFF in HP may be as high as 10%, with large numbers never being treated with antiresorptives [20]. ART is a known cause of low ALP [12] and prolonged use of ART is a known risk factor for AFF [21]. Biomarkers, such as a high urinary phosphoethanolamine level and serum PLP (main circulating form of vitamin B6) can differentiate HP from other causes of low ALP [14, 22]. It is worth noting that none of the patients in this study underwent genetic testing for HP, and it seems likely that there is an increased need for screening in this population.

Despite the differences in serum ALP, it is notable that there was no significant difference in the other hepatic indices observed between groups. To the best of our knowledge, there are no published studies comparing liver function tests (LFTs), electrolytes, coagulation profiles, or blood group in patients with AFF vs. TFF.

Higher levels of 25OHD observed in this study may reflect higher secondary prevention in the established bisphosphonate group, which has been reported previously [14]. One study has demonstrated significantly higher levels of 25OHD in patients with AFF when compared to TFFs [23], but results are conflicting [24]. However, alternative explanations merit consideration. Supplementation practices may differ systematically between groups; for example, patients with a prior fragility fracture are often targeted for high-dose vitamin D or combined calcium–vitamin D preparations in primary and secondary care. Differences in comorbidities could also contribute, as CKD, malabsorption, or cognitive impairment (all more frequent in the TFF group in our cohort) may impair vitamin D handling or adherence to supplementation. A referral bias cannot be excluded: patients with regular osteoporosis clinic follow-up, often with long-term bisphosphonate therapy, may be more likely to undergo routine monitoring and correction of vitamin D status, whereas those presenting with TFFs may be less intensively screened. Finally, the paradoxical finding that higher 25OHD is associated with AFF in some series suggests that vitamin D status may be more a surrogate marker of healthcare exposure and treatment intensity than a causal factor in fracture risk [14].

Higher levels of vitamin B12 may also reflect higher level of surveillance, although interestingly, Li et al. found that higher levels of serum B12 was a risk factor for osteoporotic vertebral fractures [25] Lower creatinine levels observed in this cohort may reflect lower levels of muscle mass (a marker of frailty) in the AFF group, as long-term bisphosphonate use is associated with a significantly smaller cross-sectional muscle mass when compared to controls [26]. Moreover, there was a higher proportion of male patients in the TFF group, which may explain discrepancies in creatinine/muscle mass and weight between groups. A larger proportion of patients with the TFF group had dementia, a known risk factor for osteoporotic fractures [27].

Studies have demonstrated an independent association between osteoporosis and lower serum albumin levels [28–30]. However, statistically significant differences in levels of albumin and globulin between groups are unlikely to be clinically significant, as the mean plasma level of both albumin and globulin observed in this study was in the normal range.

Concerningly, more than half the patients with AFF were continued on ART despite this being an absolute contraindication [3]. This indicates the need for increased awareness among clinicians prescribing ART.

This study had limitations which must be considered. The retrospective nature of the study increases the risk of selection bias, uncontrolled variables, and missing data. However, selection bias was mitigated by inclusion of all patients presenting with femoral shaft fractures. Furthermore, ALP is affected by numerous other factors, and it is not possible to infer causality. Use of ICD-10 codes is not an optimal means of identifying patients and there is a risk of missing data, but individual chart review of all femoral fractures mitigated this risk. Some of the studied parameters (B12, folate, and 25OHD) are not routinely collected in all patients and a large minority of patients did not have these measured. Unfortunately, bone-specific ALP was not measured, and prospective studies could consider employing this assay, in addition to urinary phosphoethanolamine level and serum PLP (main circulating form of vitamin B6) where a diagnosis of HP is suspected (or where ALP is < 40 IU/L).

Strengths of this study include the use of a panel of experts to adjudicate AFFs and a relatively large sample size given the low incidence of AFF. Moreover, a large proportion of patients in both groups had biochemical parameters available for examination.

This study highlights the need to raise awareness not only of AFF but of other possible mimics and to be mindful that “atypical” femoral fractures may actually be “typical” fractures where there is an underlying metabolic bone disease.

## 5. Conclusion

This study highlights a number of distinct clinical and biochemical features of patients presenting with AFFs, particularly the association with suppressed ALP and increased 25OHD. These findings suggest that low ALP levels may serve as an important diagnostic clue at the time of hospital admission. The high rate of inappropriate continuation of ART at discharge underscores a significant gap in recognition and clinical management of AFFs within acute care settings. Given the overlap with conditions such as HP, further prospective research is warranted to explore the utility of biochemical screening and to develop clinical decision support tools that guide appropriate cessation or modification of ART in this high-risk population.

## Figures and Tables

**Table 1:** The comparison by univariate analysis of admission blood results between patients presenting with AFF vs. TFF.

	Normal range	AFF (*n* = 41) No. of readings	Value (IQR) [SD]	TFF (*n* = 101) No. of readings	Value (IQR) [SD]	*p* value
ALP (U/L)	(30–110)	41	56 (41, 75)	101	83 (63.3, 107)	< 0.01
GGT (U/L)	(< 55 U)	27	45 (16, 45)	101	24 (15, 45)	0.61
ALT (U/L	(< 45)	40	20 (14, 25)	99	16 (12, 25)	0.18
AST (U/L)	(< 35)	39	22 (18, 28)	98	22 (17, 32)	0.79
Creatinine (umol/L)	(64–108)	37	68 (57, 75)	101	77 (61, 104)	0.05
Urea (mmol/L)	(2.9–8.2)	40	7.3 (5.9, 8.8)	101	8.01 (5.1, 9.4)	0.33
LDH (unit/L)	(120–250)	33	262 (226, 293)	81	269 (243, 318)	0.27
Albumin (g/L)	(35–50)	40	38 (35, 41)	101	36 (35, 41)	0.02
Globulin (g/L)	(25–45)	40	27 (25, 30)	101	29 (27, 32)	0.02
K (mmol/L)	(3.5–5.2)	39	4.13 (3.9, 4.4)	101	4.27 (3.9, 4.6))	0.41
Na (mmol/L)	(135–145)	40	137 (135, 138)	101	137 (135, 139)	0.74
Mg (mmol/L)	(0.70–1.10)	33	0.81 (0.75, 0.91)	94	0.81 (0.75, 0.87)	0.42
TSH (mU/L)	(0.4–4)	29	1.5 (0.9, 3.0)	61	2.2 (0.9, 3.6)	0.72
FT4 (pmol/L)	(11.5–22.7)	28	14 (13, 17)	59	13 (11.8, 16.3)	0.11
Vitamin B12 (pmol/L)	(133–680)	25	360 (168, 403)	64	249.4 (143, 276)	0.03
CCa2+ (mmol/L)	(2.10–2.60)	35	2.31 (2.24, 2.37)	98	2.32 (2.24, 2.38)	0.89
Phosphate (mmol/L)	(0.75–1.50)	35	1.12 (0.91, 1.3)	98	1.17 (0.96, 1.3)	0.49
25OHD (nmol/L)	(> 49)	29	86.8 [29.4]	70	69.5 [27.5]	0.01
INR	(0.9–1.2)	27	1 (1.0, 1.05)	74	1.05 (1.0, 1.1)	0.02
PT (s)	(9–13)	27	12 (11, 14)	74	12 (11, 14)	0.26
Platelets (× 10^9^/L)	(140–400)	28	244 [72.8]	87	226 [75.3]	0.27

*Note:* Mean (SD), comparisons are by *t*-test; median [(Q1, Q3) where Q1 is the 1^st^ quartile and Q3 is the 3^rd^ quartile], comparisons are by the rank-sum test. IQR = interquartile range, ALP = alkaline phosphatase, ALT = alanine aminotransferase, AST = aspartate aminotransferase, LDH = lactate dehydrogenase, Ft4 = free thyroxine, .

Abbreviations: GGT = gamma-glutaryl transferase, INR = international normalized ratio, PT = prothrombin time, TSH = thyroid-stimulating hormone.

**Table 2:** The blood group frequencies between TFFs and AFFs.

Blood group	TFF group	Frequency (%)	AFF group	Frequency (%)
	*N* = 101		*N* = 41	
A+	38	37.255	19	45.238
A−	2	1.961	2	4.762
AB+	1	0.980	2	4.762
B+	10	9.804	2	4.762
B−	1	0.980	2	4.762
O+	35	35.294	9	23.810
O−	9	8.824	3	7.143
(Missing)	5	4.902	2	4.762

**Table 3:** The clinical characteristics, comorbidities, and medications between the AFF and TFF groups.

	AFF (*n* = 43)	TFF (*n* = 101)	*p* value
*Clinical characteristics*			
Male	4 (9.3%)	24 (23.8%)	0.04
Female	39 (90.7%)	76 (75.2%)	
Age (years) [SD]	73.7 [11]	76 [14.8]	0.04
Weight (kg) [SD]	64.5 [14.9]	72.8 [18.4]	0.02
BMI (kg/m^2^) [SD]	25.4 [6]	27 [6.7]	0.14
Length of stay (days) [SD]	11 [8.8]	16.6 [17.3]	0.25
Previous fracture	23 (53.5%)	27 (26.7%)	< 0.01
Prodrome	14 (32.6%)	3 (3%)	< 0.01

*Comorbidities*			
Osteoporosis	42 (97.7%)	36 (35.6%)	< 0.01
Dementia	3 (7%)	25 (24.8%)	< 0.01
Rheumatological disease	11 (25.6%)	12 (11.9%)	0.30
COPD	8 (18.6%)	18 (18.6%)	0.99
CKD	3 (7%)	14 (13.9%)	0.39

*Medications*			
Bisphosphonate	29 (67.4%)	6 (5.9%)	< 0.01
Average duration (years) [SD]	10.8 (5.5)	3.4 (1.7)	< 0.01
Denosumab	17 (39.5%)	17 (16.8%)	< 0.01
25OHD (nmol/L)	17 (39.5%)	36 (35.6%)	0.45
Average duration (years) [SD]	4 [2.9]	2.6 [1.7]	0.19
Proton pump inhibitor	23 (53.5%)	37 (36.6%)	0.06
Antidepressants	15 (34.9%)	33 (32.7%)	0.85
Inhaled corticosteroids	6 (14%)	13 (12.9%)	0.79
Long-term steroid use	14 (32.6%)	16 (15.8%)	0.03
Thyroxine	4 (9.3%)	7 (6.9%)	0.73

*Note:* Mean [SD], comparisons are by *t*-test. Frequencies are demonstrated using (%), 25OHD = 25-hydroxyvitamin D.

Abbreviations: CKD = chronic kidney disease, COPD = chronic obstructive pulmonary disease.

## Data Availability

Deidentified patient data underlying this article will be shared on reasonable request to the corresponding author.
